# Improved Stability and Catalytic Efficiency of ω-Transaminase in Aqueous Mixture of Deep Eutectic Solvents

**DOI:** 10.3390/molecules28093895

**Published:** 2023-05-05

**Authors:** Hongpeng Wang, Mercy Vimbai Masuku, Yachen Tao, Jiayao Yang, Yi Kuang, Changjiang Lyu, Jun Huang, Shengxiang Yang

**Affiliations:** 1 Zhejiang Provincial Key Lab for Chemical and Biological Processing Technology of Farm Product, School of Biological and Chemical Engineering, Zhejiang University of Science and Technology, Hangzhou 310023, Chinajiayao_yang@163.com (J.Y.);; 2 Zhejiang Provincial Key Laboratory of Chemical Utilization of Forestry Biomass, Zhejiang A&F University, Hangzhou 311300, China

**Keywords:** catalytic efficiency, deep eutectic solvents, thermal stability, molecular docking

## Abstract

The efficient biosynthesis of chiral amines at an industrial scale to meet the high demand from industries that require chiral amines as precursors is challenging due to the poor stability and low catalytic efficiency of ω-transaminases (ω-TAs). Herein, this study adopted a green and efficient solvent engineering method to explore the effects of various aqueous solutions of deep eutectic solvents (DESs) as cosolvents on the catalytic efficiency and stability of ω-TA. Binary- and ternary-based DESs were used as cosolvents in enhancing the catalytic activity and stability of a ω-TA variant from *Aspergillus terreus* (E133A). The enzyme exhibited a higher catalytic activity in a ternary-based DES that was 2.4-fold higher than in conventional buffer. Moreover, the thermal stability was enhanced by a magnitude of 2.7, with an improvement in storage stability. Molecular docking studies illustrated that the most potent DES established strong hydrogen bond interactions with the enzyme’s amino acid, which enhanced the catalytic efficiency and improved the stability of the ω-TA. Molecular docking is essential in designing DESs for a specific enzyme.

## 1. Introduction

Chiral amines are indispensable precursors of pharmaceutical drugs and agrochemicals; hence, they are in high demand. Among the enzymes used in the biosynthesis of chiral amines, ω-transaminases (ω-TAs) have been reported as the most potent [1]. Recently, the employment of ω-TAs at an industrial scale has attracted great attention in pharmaceutical and agrochemical industries [2,3,4,5,6,7]. However, the main obstacles inherent to ω-TAs are their poor stability and low catalytic efficiency, which limits the implementation of ω-TAs at an industrial-scale [1,8]. To overcome these obstacles, protein engineering and enzyme immobilisation are often used, although there are some problems, such as lengthy reaction times, being costly and imparting a negative influence on the environment [9]. Therefore, it is highly attractive yet challenging to improve the catalytic activity and stability of ω-TAs using greener and more efficient methods.

Solvent engineering has proven to be a promising strategy to enhance the product yield and stability of enzymes [10]. The ability of a solvent system to preserve enzyme stability, enhance catalytic efficiency and improve substrate dissolution with limited detrimental effects on the environment are some of the attractive features required in designing novel green solvents for biocatalysis. Deep eutectic solvents (DESs) have proven to be potent green alternative solvents harbouring impeccable characteristics that favour efficient biocatalysis [11,12,13]. DESs are generally prepared by thermal mixing of an ammonium salt, such as choline chloride (ChCl), and a hydrogen bond donor (HBD) to attain a eutectic mixture characterised by a melting point depression relative to the distinctive constituents [14,15]. The characteristics of DESs can be tweaked to suit the desired application by varying the constituents of the DESs.

Owing to the low production cost, ease of preparation, biodegradability, biocompatibility and nontoxicity of DESs, several reports have demonstrated the efficacy of ChCl-based DESs in biocatalysis [16,17,18,19]. The stability, selectivity and enzyme activities of lipases, peroxidases, hydrolases and proteases are preserved when free enzymes and immobilised enzymes, as well as whole cells, are used in biocatalysis [19,20,21,22]. Most of the enzymes have been found to be more active when DESs are used as cosolvents rather than pure solvents [22,23,24,25,26]. However, there are few reports on the enhancement of the catalytic efficiency and stability of ω-TAs in deep eutectic solvents.

In this study, the efficacy of aqueous solutions of binary-based DESs and ternary-based DESs (10 in total) as cosolvents on the enzymatic activity of a ω-TA variant from *Aspergillus terreus* (E133A) was examined. In addition, the thermal stability and storage stability of the enzyme in the two most potent DESs were determined. The ternary-based DES (ChCl:ethylene glycol:1,2-propanediol) enhanced the ω-TA activity by 240.5%, and the thermal stability improved 2.7-fold in contrast to the conventional buffer. Moreover, the intrinsic molecular interaction between the most potent DES and the enzyme during transamination was elucidated through molecular docking. The use of DESs as cosolvents in the asymmetric synthesis of chiral amines is a promising sustainable strategy in enhancing ω-TA activity and preserving its stability.

## 2. Results and Discussion

### 2.1. Screening of Various DESs in Enzyme-Catalysed Reaction

**Scheme 1:**
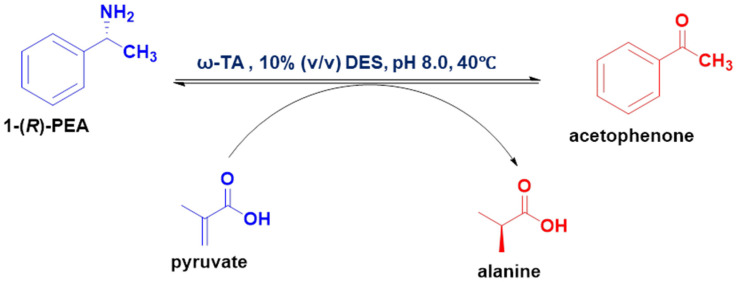
The production of acetophenone from 1-(*R*)-PEA.

**Figure 1:**
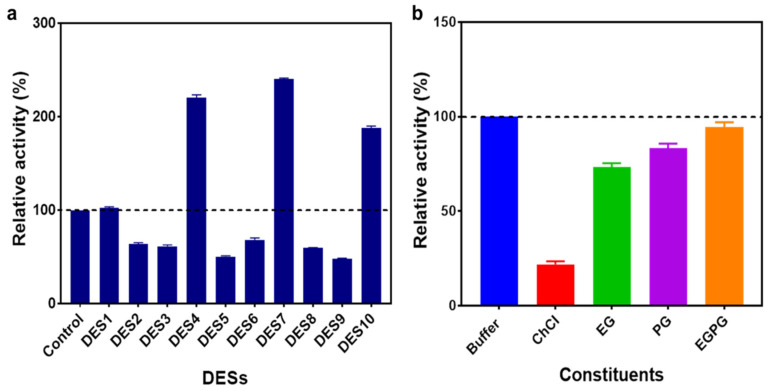
(**a**) The relative activities of the enzyme in aqueous solutions of DESs (10% (*v*/*v*)). (**b**) The relative activities of the enzyme in 10% (*v*/*v*) aqueous solutions of ChCl, EG, PG and EGPG. The reaction that was conducted in DES-free buffer was considered the control, and the relative activity was taken to be 100%.

The influence of 10 aqueous solutions of DESs (Table 1) as cosolvents on the ω-TA (E133A) was evaluated. The ω-TA activity in the aqueous solutions of the DESs (Scheme 1) was compared with the activity in the DES-free phosphate buffer (pH 8.0). The enzyme activity varied depending on the type of DES used, as illustrated in Figure 1a. Among the screened DESs, the ω-TA activity was either enhanced or preserved in only four of the aqueous DES solutions. The rest of the DESs induced a decline in enzyme activity. Overall, the alcohol-based DESs (containing glycerol, ethylene glycol (EG) and 1,2-propanediol (PG)) as HBDs were more potent in enhancing the activity of the enzyme relative to the reaction in phosphate buffer. However, some of the DESs containing HBDs with hydroxyl groups had relative activities lower than the control, which could have been influenced by the HBDs forming interactions with the product, acetophenone (Scheme 1), thereby restraining the release of the product from the domain of the enzyme [24].

The relative activity of the ω-TA in DES4 (binary-based DES) was 220%, 240.5% in DES7 (ternary-based DES) and 188% in DES10 (ternary-based DES). The aqueous solution of the ternary-based DES (DES7), which contained hydroxyl groups as the hydrogen bond donor, elevated the activity of the ω-TA variant to a greater extent, with a relative activity of 240%. The presence of more hydroxyl groups in DES7 denoted an enhanced polarity and hydrogen bond network, which contributed to the stabilisation of the tertiary structure of the ω-TA, hence, an enhanced activity [27,28]. Even though three of the aqueous solutions of the alcohol-based DESs (DES4, DES7 and DES10) exhibited enhanced activity, the other aqueous solutions of the alcohol-based DESs induced a decline in the ω-TA activity irrespective of the presence of the hydroxyl groups. The formation of side reactions between the substrate; 1-(*R*)-phenylethylamine (1-(*R*)-PEA) and the DESs could have influenced the decrease in activity as a result of the substrate being trapped within the DES system, hence failing to participate in the reaction. Furthermore, the type of HBDs and the composition in a DES system have an influence on the enzyme activity due to the differences in the complexity of the formed hydrogen bond network [24,29].

An increase in the molar ratio of HBDs in aqueous solutions of the amide-based DESs (DES1-DES3) triggered a decline in the enzymatic activity. We, therefore, ascertained that increasing the molar ratio of urea adversely influences the enzyme activity. The relative activity of the ω-TA decreased from 104% (DES1) to 61% (DES3). Nevertheless, the inhibitory effect was almost negligible from DES2 to DES3, indicating maintenance of the tertiary structure of the enzyme; hence, we disregarded enzyme denaturation as the cause of the decrease in the enzyme activity. The destabilisation of the enzyme–substrate complex, such as the external aldimine formed upon binding of the substrate to the internal aldimine in the amide-based DESs, could have induced a decline in the ω-TA activity. The amide-based DESs were, hence, poor at improving the activity of the ω-TA variant.

The aqueous solution of the ternary-based DES with EG and PG as the hydrogen bond partners was excellent at enhancing the ω-TA activity. Based on the results observed for the first screening, the aqueous solutions of a binary-based DES (DES4) and ternary-based DES (DES7) that exhibited a significant improvement in the ω-TA activity were selected for further investigations. To further ascertain if the enhancement in enzyme activity was a result of the two aqueous DES solutions (exhibiting the highest activity) or individual constituents comprising the DES, the relative activities of the aqueous solutions (10% (*v*/*v*)) of the individual constituents of the DES were further analysed and compared with the activity in the DES system.

The relative activities of the transaminase in the aqueous solutions of the most potent DESs (DES4 and DES7) demonstrated a more pronounced increment in activity over the individual DES constituents. As depicted in Figure 1b, the relative activities of the aqueous solutions of the individual constituents and the DESs with increasing order were as follows: ChCl < EG < PG < ethylene glycol:1,2-propanediol (EGPG) < phosphate buffer < DES4 < DES7. The relative activity of the transaminase was significantly lower in aqueous ChCl (<25%). ChCl, thus, deactivated the enzyme, as it had the least relative activity in comparison with the EG and PG. The low activity from the individual constituents demonstrated that the enhancement of enzyme activity was a result of the DES system rather than the individual constituents. The negative impacts of the ChCl, EG and PG are circumvented when the constituents form a DES complex in aqueous solution. EG has been found to act as a competitive enzyme inhibitor in biocatalysis, which does not occur when it is incorporated as a DES (for instance, ChCl:EG) [27,30].

Notably, when EG and PG were mixed to form a DES-like system, the activity of the enzyme was lower than the activity in the conventional buffer, which indicates that ChCl plays a crucial role in enhancing enzyme activity when it is in a DES complex with the hydrogen bond donors. The interactions that were formed by ChCl and the hydrogen bond donors facilitated an improvement in the ω-TA activity as opposed to either using the individual DES components or combining the hydrogen bond donors without the ChCl.

### 2.2. The Effect of DES Concentration on Enzyme Activity

**Figure 2:**
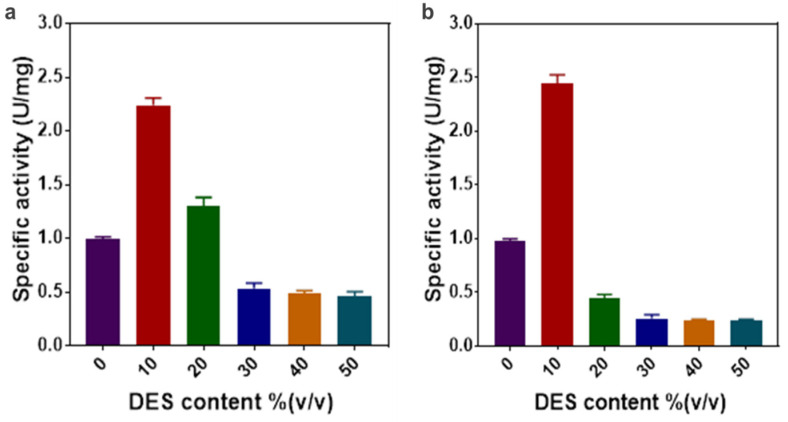
(**a**) The effect of the concentration of the aqueous solution of DES4 on the specific activity of the enzyme. (**b**) The effect of the concentration of the aqueous solution of DES7 on the specific activity of the enzyme.

The impact of the aqueous solutions of a DES4 and DES7 concentration on the activity of the ω-TA was investigated. In regard to the specific activity, the highest ω-TA activity was achieved in 10% (*v*/*v*) DES7 with a specific activity of 2.4 U mg^−1^; hence, the activity was enhanced by an increment of 2.4 (Figure 2). Nevertheless, an elevated enzyme activity was also observed at 10 and 20% (*v*/*v*) DES4 with specific activities of 2.2 U mg^−1^ and 1.3 U mg^−1^, respectively. A drastic decrease in the enzyme activity was observed at increasing DES concentrations. Notably, the enzyme was more susceptible to an increase in the DES7 concentration in contrast to the DES4 concentration. An increase in the DES concentration was directly proportional to the viscosity. High viscosities have an influence on the specific activity and stability of enzymes [24].

At high DES concentrations, the decrease in the enzyme activity can be attributed to the high viscosity of the DES resulting from the extensive hydrogen bonds, van der Waals forces and electrostatic interactions within the DESs [18]. The extensive hydrogen bonding network that formed within the DES, in turn, reduced the mass transfer of the reaction intermediates, thereby reducing the enzyme activity [27]. The substrate 1-(*R*)-PEA and the cosubstrate pyruvate could have been restrained from moving spontaneously at the protein core as a result of the formation of an extensive hydrogen bond network between the DES and the surface amino residues of the enzyme [31,32,33]—hence, limiting efficient catalysis. Furthermore, a high DES concentration could have caused the destabilisation of the reaction intermediate complexes. Therefore, a high DES content has an impact on the transaminase activity due to the subversion of the reaction intermediates.

### 2.3. The Effect of pH and Temperature on Enzyme Activity

**Figure 3:**
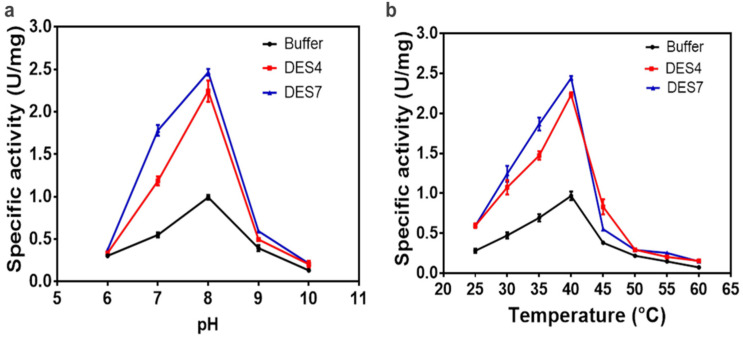
(**a**) The effect of pH on the specific activity of the enzyme in aqueous solutions of DES4 and DES7. (**b**) The effect of temperature on the specific activity of the enzyme in aqueous solutions of DES4 and DES7. The experiment was conducted in triplicate and run in parallel with the control (phosphate buffer). Black: buffer; red: DES4; blue: DES7.

To further understand the enzymatic properties of the ω-TA in aqueous solutions of DES4 and DES7, we investigated the effect of the reaction temperature and pH on the enzyme activity. As illustrated in Figure 3, the optimal temperature and pH of the ω-TA were 40 °C and pH 8.0, respectively, in the two aqueous DESs. There was a steep decline in the enzyme activity when the temperature was elevated above 40 °C, indicating the susceptibility of the enzyme to thermal inactivation at temperatures greater than 40 °C regardless of the solvent used. The optimum reaction conditions in conventional buffer and in the aqueous solutions of the DESs were comparable.

The investigated aqueous DES solutions had a negligible impact on the enzyme’s tolerance to temperature and pH. Although the results for the different reaction media showed comparable trends, DES7 seemed to induce a tolerance of pH 7.0, more than the conventional buffer and DES4. Overall, the DESs did not partake in stabilising the transaminase variant to tolerate an extreme pH and elevated temperature, as extreme reaction conditions inactivated the enzyme.

### 2.4. Optimisation of Reaction Conditions Using Response Surface Method

**Figure 4:**
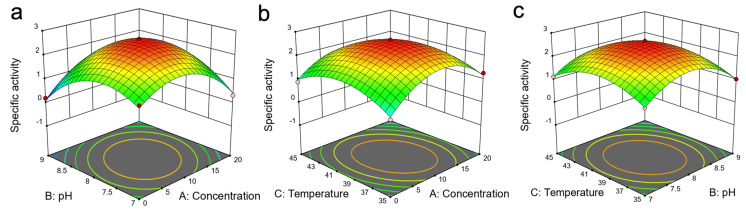
The 3D response surface plots showing the relationship of the three variables (concentration, pH and temperature) on the specific activity of the enzyme in aqueous solution of DES7: (**a**) effect of pH and DES concentration on specific activity of the enzyme; (**b**) effect of temperature and DES concentration on specific activity of the enzyme; (**c**) effect of temperature and pH on specific effect of the enzyme.

In order to ascertain the optimum reaction conditions for the ω-TA in aqueous solutions of DES7, a quadratic response surface was determined using Box–Behnken by testing three variables in a single experiment. The correlations of the DES concentration, pH, and reaction temperature of the enzyme activity, subsequent to an ANOVA analysis of the results of the experimental data (Table S1) and the predicted values, were plotted as 3D response surface plots (Figure 4). An ANOVA analysis was implemented to confirm the adequacy of the model (Table S2). A low *p*-value (*p* < 0.0001) and a high F-value (88.63) indicate the significance of the model. The *p*-value and the F-value of the lack-of-fit were 0.112 and 3.86, respectively, which demonstrates the insignificant difference of the lack-of-fit relative to the pure error. A good relevance of the variables was indicated by a coefficient of determination (*R*^2^) close to 1 (*R*^2^ = 0.991). There was a good correlation between the predicted values and actual values of the enzyme activity, as illustrated by an adjusted determination coefficient (*R*^2^_adj_) of the model of 0.980. The signal-to-noise ratio was measured by Adeq precision, which should be greater than 4. In this model, the Adeq precision was 26.4, which denotes that the model could be used to navigate the design spaces.

Notably, the optimal reaction conditions were as follows: pH 7.9, a reaction temperature of 39 °C and 9.8% (*v*/*v*) DES concentration. The response surface method, thus, further verified the optimum reaction conditions that created a more favourable microenvironment at the catalytic active site of the enzyme. The specific activity of the enzyme at these optimised reaction conditions was 2.67 U mg^−1^. The results from single factor or single variable tests were in coherence with the results from response surface method.

### 2.5. Determination of the Kinetic Parameters in the Presence of DESs

The kinetic parameters of the ω-T in the aqueous solutions of DES4 and DES7 and the DES constituents were determined, as illustrated in Table 2 and Figures S1 and S2 (Supplementary Materials). The experimental results were evaluated using the Michaelis–Menten model to validate the influence of the aqueous solutions on transaminase-catalysed reaction with pyruvate and 1-(*R*)-PEA as the substrates. When the concentration of pyruvate was kept constant and the concentration of 1-(*R*)-PEA was varied, the largest *K*_m_ value (0.65 ± 0.07 mM) was recorded when the phosphate buffer was used, while the lowest *K_m_* value of 0.48 ± 0.02 mM was obtained in the presence of DES7. Furthermore, the *K_m_* of the enzyme decreased to 0.52 ± 0.04 mM in DES4. We, therefore, conclude that the presence of 10% (*v*/*v*) of the DESs accelerated the stimulation of the ω-TA, which was indicated by the lower *K_m_* values. There was a more pronounced binding affinity of 1-(*R)*-PEA towards the ω-TA variant in the two aqueous solutions of the DESs contrary to the phosphate buffer. The same trend in the results was obtained when the concentration of pyruvate was varied.

Herein, we hypothesised that the role of the DES was to interact with the enzyme in a manner that stabilised the protein rather than inhibiting the enzyme, which was supported by an improvement in the turnover number (*k_cat_*) and catalytic efficiency (*k_cat_/K_m_*). The interaction promoted the positioning of the amino group of 1-(*R*)-PEA towards the internal aldimine, hence, inducing an increment in the substrate affinity [34]. The decrease in *K_m_* indicated that the interactions formed by the aqueous solution of DES7 did not induce competitive inhibition with the substrate, as some DESs can compete with the substrate to bind at the catalytic amino acid residue thereby restraining the binding of the substrate [27,30].

The catalytic efficiency of the enzyme was enhanced three-fold relative to the DES-free buffer. Notably, there was a small deviation in the catalytic efficiency of the ω-TA in DES4 and DES7, since in DES4 the catalytic efficiency was enhanced 2.5-fold. The interactions formed between DES7 and the key amino acid residues in the enzyme were, hence, stronger than the interactions formed between DES4 and the key amino acid residues of the enzyme. The deviation in the *K_m_*, *k_cat_* and *k_cat_*/*K_m_* in the binary and ternary DESs demonstrated the higher enzyme activity in DES7 in contrast to DES4.

On the other hand, when the aqueous solutions of ChCl, EG, PG and EGPG were used, the *K_m_* values were all higher than the *K_m_* value of the enzyme in the phosphate buffer or aqueous DES solutions. We, therefore, concluded that the strong substrate affinity of the enzyme in the aqueous DES solutions was a result of the combined effect of the ChCl and the HBDs. When either ChCl or the HBDs were used in the reaction, the affinity of the enzyme towards the substrate was reduced. In addition, both the turnover number and catalytic efficiency of the enzyme were reduced when the individual DES constituents were used in the reaction system. These results indicate that the interactions formed within the aqueous solutions of DES are critical to improving the ω-TA activity.

### 2.6. Stability of the ω-Transaminase in Aqueous Solutions of DES

**Figure 5:**
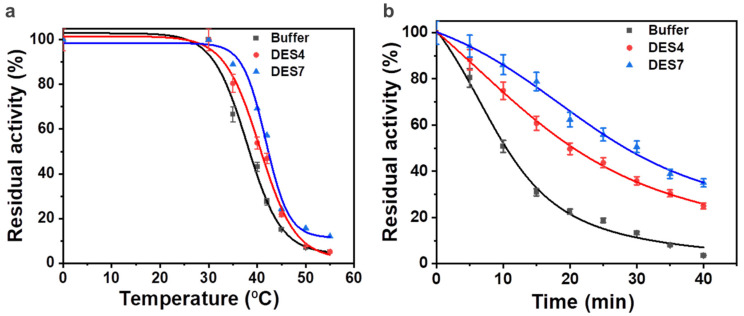
The thermal stability of the enzyme in aqueous solutions of DES4 and DES7: (**a**) inactivation temperatures (*T*_50_) of the enzyme in buffer (black), DES4 (red) and DES7 (blue); (**b**) half-lives of the enzyme in buffer (black), DES4 (red) and DES7 (blue) at 40 °C.

To achieve comprehensive insight into the influence of the aqueous solutions of the binary-based DES and ternary-based DES on the thermal stability of the enzyme, the half-lives and the deactivation temperature of the enzyme in the aqueous DES solutions were assessed. In the presence of 10% (*v*/*v*) DES4 at 40 °C, the deactivation of the ω-TA was slower than the deactivation in phosphate buffer, as illustrated by an increment in the half-life (*t_½_*) from 10.49 min to 20.57 min (Figure 5 and Table 3). DES7 enhanced the thermal stability of the ω-TA to a greater extent (*t*_½_ = 28.36 min), thus achieving a half-life 2.7 times longer than in the conventional buffer.

Compared with the inactivation temperature (*T*_50_) in phosphate buffer (38.0 °C), DES4 (40.8 °C) and DES7 (42.2 °C) showed an improvement in the thermal stability. The inactivation temperature was almost in agreement with the results obtained when the temperature was tested as a single variable. An increase in temperature above 42 °C induced the disintegration of the catalytic active site of the transaminase, which was indicated by the loss of the enzyme activity. The aqueous solution of DES7, thus, improved the thermal stability of the ω-TA more than DES4 and phosphate buffer.

The ternary-based DES consisting of hydroxyl functional groups induced a higher half-life, which was 2.7-fold higher than the half-life in the conventional buffer. The inactivation of the enzyme was slower in both the binary and ternary alcohol-based DES solutions compared to the conventional buffer, demonstrating the efficacy of the hydroxyl functional groups at stabilising the enzyme. The enzyme was stabilised by the intermolecular hydrogen bond interactions formed by DES. The enhanced thermal stability observed in DES7 could have been due to the presence of stronger intermolecular hydrogen bond interactions due to the additional hydroxyl groups than in DES4.

### 2.7. Molecular Docking Simulation

**Figure 6:**
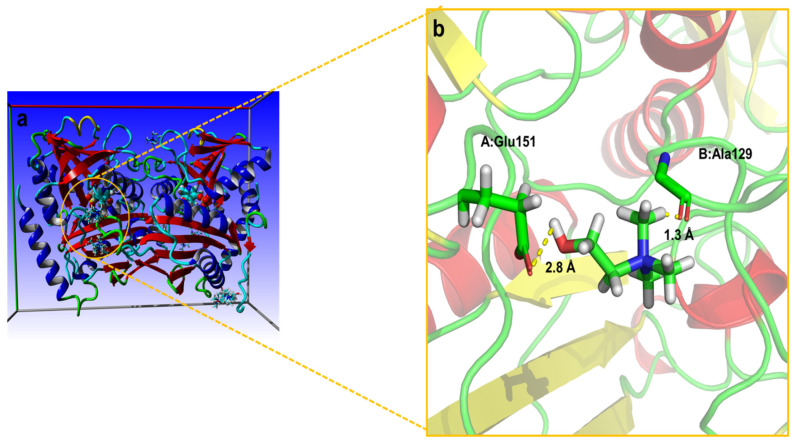
Molecular docking of the enzyme with ChCl: (**a**) distribution of ChCl around the protein; (**b**) hydrogen bond interactions between the enzyme and ChCl near the pocket.

**Figure 7:**
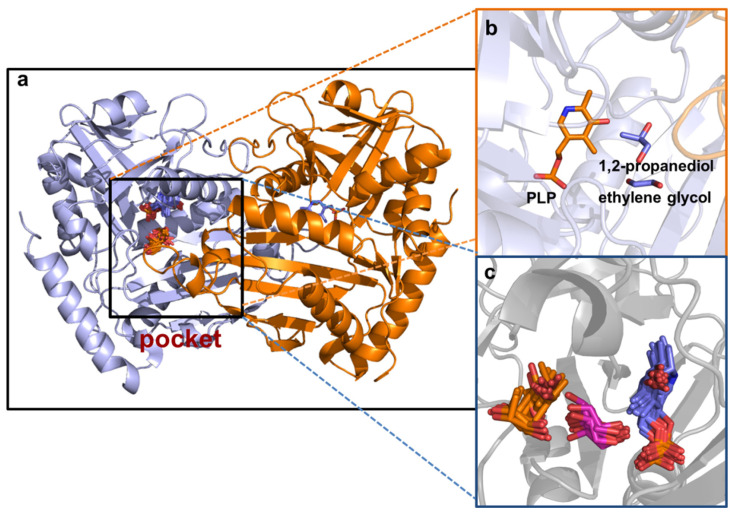
The molecular docking of the enzyme with EG and PG: (**a**) pocket of the enzyme; (**b**) location of the DES constituents and PLP in the pocket with a docking affinity energy of −18.4 kcal mol^−1^; (**c**) all possible docking positions of PLP, EG and PG.

A molecular docking analysis was performed to illustrate the preferential intrinsic molecular interactions of the most potent aqueous DES solution (DES7) with the ω-TA, which contributed to an improvement in the catalytic efficiency and stabilisation of the ω-TA. ChCl was absent in the pocket of the enzyme, and EG and PG were the only DES constituents located in the pocket of the enzyme (Figure 6 and Figure 7). The ChCl, thus, could not migrate to the protein domain as a result of low diffusion coefficients. The ChCl merely formed some hydrogen bonds with residues (Glu151 and Ala129) on the surface of the enzyme, as depicted in Figure 6. ChCl, thus, did not interact with the catalytic amino acid residues at the protein. When ChCl formed the hydrogen bonds with Glu151 and Ala129 on the surface of the protein, it could have disrupted some intramolecular hydrogen bonds, which triggered a decline in the enzyme activity when the aqueous solution of ChCl was used in the enzyme activity assay.

The other two constituents of the ternary DES, EG and PG, with the capacity to migrate to the protein domain were in proximity with the coenzyme pyridoxal-5’-phosphate (PLP) (Figure 7). The docking affinity energies and the type of interactions formed by the HBDs and the key amino residues in the domain of the enzyme are shown in Table 4. Molecular diagrams illustrating the intrinsic molecular interactions are shown in Figure S3 (Supplementary Materials). There were four interactions (two conventional hydrogen bonds and two carbon hydrogen bonds) between the HBD EG and the key amino acid residues in the pocket of the enzyme. The EG formed two conventional hydrogen bonds with Lys180 (2.9 Å) and Tyr60 (2.0 Å) and carbon hydrogen bonds with Val62 (2.9 Å) and His55 (2.2 Å). Contrarily, PG formed two conventional hydrogen bonds with His55 (2.1 Å) and Tyr60 (2.2 Å).

Although PG formed two interactions with the amino acid residues in the pocket, these interactions were stronger than the four interactions formed by EG. This was illustrated by the large docking affinity energy of −11.4 kcal mol^−1^ in PG in contrast to EG, which had a docking affinity energy of −9.5 kcal mol^−1^ (Table 4). A large negative docking affinity energy and short bond distance denotes a stronger interaction between the HBD and the amino acid residues [35]. In this case, the bond lengths for the interaction of PG with the amino acid residues were shorter than the bond lengths for the interaction of EG with the amino acid residues, supporting that the HBD PG formed stronger interactions with the amino acid residues. We hypothesised that the hydrogen bonding formed facilitated the efficient orientation of the substrate in a manner that promoted efficient interaction among the reaction intermediates.

We deduced that the presence of EG in the DES system enhanced the affinity of the ω-TA towards the substrate, as the EG formed hydrogen bonding with the catalytic Lys180, which is involved in the reprotonation of the reaction intermediate complexes during the transamination reaction [33]. By comparing the molecular docking simulation results and the experimental results, we concluded that the effect of the potent DES emanated from the combined effect of the DES as a whole opposed to the individual constituents. The beneficial effect towards the enzyme stabilisation and enhancement of the catalytic efficiency was a result of the interactions formed by the DES components and the enzyme. The synergetic effects of the interactions formed by the ChCl and HBDs on the ω-TA attributed to an improved enzyme activity and enzyme stability, which can be justified by the low enzyme activity when EG, PG or EGPG were used in the enzyme assays.

### 2.8. Storage Stability

**Figure 8:**
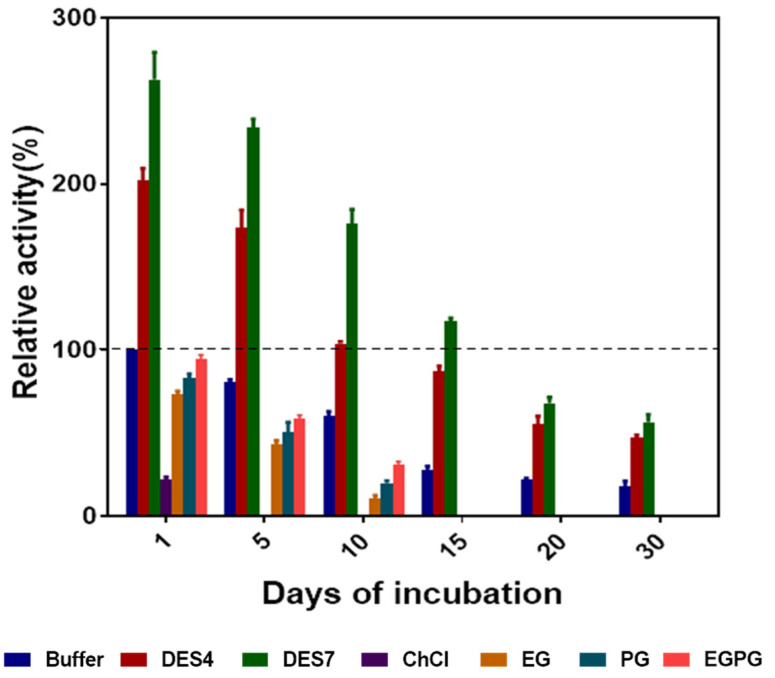
The relative activity of the enzyme after incubation in aqueous solutions of DES4, DES7, ChCl, EG, PG and EGPG for several days. The initial activity of the enzyme in buffer on the first day of incubation was taken as 100%.

The enzyme was stored at 4 °C in 10% (*v*/*v*) DES4, DES7, ChCl, EG, PG, EGPG and 50 mM phosphate buffer for a predetermined time to ascertain the effect of the DESs on the prolonged storage. The relative activity of the enzyme (with respect to the control) after storage was determined after several days. The storage stability in the aqueous solutions of the alcohol-based DESs was remarkable. As illustrated in Figure 8, the activity of the enzyme was maintained to a greater extent in DES7, as the relative activity was above 100% after 15 days of storage. However, DES4 retained the enzyme activity (above the control) up to 10 days of storage. This can be accounted for by the presence of stronger hydrogen bond interactions in DES7 than in DES4, as illustrated from the molecular docking studies. The DESs that contained hydroxyl groups are, thus, excellent enzyme stabilisers. Oh and colleagues [28] also demonstrated the efficacy of hydroxyl-based DESs in enhancing the stability of enzymes.

However, the DES constituents were poor at preserving the enzyme activity. ChCl lost all of its activity on day 5 of storage, while EG, PG and EGPG lost all of its activity on day 15. Since the activity of the enzyme in the aqueous solutions of ChCl, EG, PG and EGPG was lower than in the conventional buffer, we concluded that the stability of the enzyme in the aqueous solutions of the DES was due to the fact of hydrogen bond interactions that formed within the DES system with the enzyme, which were absent in the aqueous solutions of either ChCl or hydrogen bond donors EG and PG.

## 3. Materials and Methods

### 3.1. Materials

Yeast extract and tryptone were purchased from Oxoid (Shanghai, China). Pyridoxal-5’-phosphate (PLP), pyruvate and R-1-phenylethylamine (1-(*R*)-PEA) were purchased from Aladdin Biochemical Technology Co., Ltd. (Shanghai, China). Choline chloride (ChCl), urea, ethylene glycol (EG), propylene glycol (PG), glycerol, formate, kanamycin sulphate, isopropyl–β-d-thiogalactopyranoside (IPTG), sodium dihydrogen phosphate, disodium phosphate, imidazole, sodium chloride, Ni-NTA Sefinose™ Resin (Settled Resin), Modified Bradford Protein Assay kit and SDS PAGE gel kit were obtained from Sangon (Shanghai, China). Amicon^®®^ Ultra Filter units were obtained from Sigma Aldrich (Shanghai, China). All chemicals were of analytical grade. The transaminase genes from *Aspergillus terreus*, which were previously transformed in *E. coli* BL21 (DE3) pRSF-D-AAO competent cells, were stored in our laboratory at −80 °C. The plasmids and the *E. coli* BL21 (DE3) pRSF-D-AAO competent cells were constructed by Anhui General Biology Company (Anhui, China).

### 3.2. Methods

All experiments were conducted in triplicate, and the results are given as the mean ± standard deviation. The tubes with the aqueous DES solutions and substrates were the blanks of the experiments.

#### 3.2.1. DES Preparation

The 10 DESs were prepared by thermal mixing of the hydrogen bond acceptor (HBA) ChCl and various hydrogen bond donors (HBDs) at varying molar ratios (as illustrated in Table 1) with the aid of a magnetic stirrer at 80 °C for 2–3 h to achieve a homogenous, clear and colourless liquid. The DESs were sealed and stored at room temperature. The aqueous solutions of the various DESs were prepared by mixing each DES with 50 mM phosphate buffer at pH 8.0 to make a final volume/volume percentage of 10% (*v*/*v*).

#### 3.2.2. Induction and Purification of ω-Transaminase

The transformants harbouring the recombinant target gene E133A [36] were grown in LB media containing 50 µg/mL kanamycin at 37 °C to achieve an OD_600_ of 600–800. Thereafter, the culture was induced with 1 mM IPTG (final concentration) and further grown at 25 °C, 150 rpm for 20 h. The cells were harvested, resuspended in binding buffer (300 mM NaCl, 50 mM phosphate buffer and 20 mM imidazole, pH 8.0) and disrupted with a high-pressure homogeniser at 4 °C, 700 psi for 3 min. The cell lysate was centrifuged prior to purification on a Ni-NTA affinity column. Subsequently, the concentration and pre-treatment of the protein was conducted through ultrafiltration with Amicon^®®^ Ultra-0.5 centrifugal units (MWCO 10 kDa) using 10% (*v*/*v*) DES. The determination of the protein concentration and protein purity was performed using the Bradford protein assay (Bio-Rad, Beckman Coulter, Brea, CA, USA) and 12% SDS-PAGE, respectively. 

#### 3.2.3. Screening of DESs in ω-Transaminase-Catalysed Reactions

To examine the influence of the 18 DESs on the ω-TA, E133A, the purified enzyme was subjected to a 3 min enzyme activity assay. The reaction was initiated by the addition of 20 µL of the enzyme/DES solution (0.2 mg/mL) to 180 µL of substrate (2.5 mM 1-(*R)*-PEA, 2.5 mM pyruvate and 0.1 mM PLP in 10% (*v*/*v*) DES, pH 8) for 3 min at 40 °C (Scheme 1) and 200 rpm in a thermomixer (Eppendorf Thermomixer Comfort, Eppendorf China Co., Ltd., Shanghai, China). The termination of the reaction was conducted by boiling for 5 min. All reactions were performed in triplicate. One unit of enzyme was defined as the quantity of enzyme that catalysed the production of 1 µmol of acetophenone in a min with 1-(*R*)-PEA and pyruvate. The quantification of the acetophenone produced was performed using HPLC. The specific activity of the enzyme was defined as the enzymatic activity per milligram of the protein.

#### 3.2.4. Activity of ω-Transaminase in Various Aqueous Solutions of DES Concentrations

Based on the results from the initial screening of the DESs, the aqueous solutions of DES4 and DES7 were selected for further investigations in the transamination reaction. The transaminase activity was evaluated in the aqueous solutions of the DESs with varying concentrations (0–50% (*v*/*v*)).

#### 3.2.5. Factors Influencing ω-Transaminase Activity in the Presence of Aqueous Solutions of DES

The influence of the pH and temperature on the ω-TA in the presence of the DESs was investigated by determining the enzyme activity at various pH values (6.0–10.0) and various temperatures (30–60 °C). The relative activity of the transaminase was expressed as the percentage (%) activity in 10% (*v*/*v*) DES in reference to the activity in phosphate buffer.

The precise optimum reaction conditions were determined by implementing a response surface methodology (RSM), as previously described [37,38]. The statistical design and analysis of the ω-TA activity in response to the combined three variables (i.e., DES concentration, pH and temperature) were evaluated using a Box–Behnken statistical design in Design Expert version 8.0.6 (Stat-Ease, Inc., Minneapolis, MN, USA) to achieve a second-degree polynomial model. The second-degree polynomial model was used to optimise the transamination reaction by carrying out 17 experimental runs. The correlation between the experimental data and the predicted values was analysed using ANOVA analysis.

#### 3.2.6. Kinetics and Thermal Stability of ω-Transaminase

The determination of the rate of ω-TA activity in the DESs was conducted by varying the concentration of either 1-(*R*)-PEA (0–3 mM) or pyruvate. The reaction was performed at the optimum temperature and pH, as previously described in Section 3.2.5. Furthermore, the specific activities obtained were fitted in the Michaelis–Menten equation in Origin 8.0 to obtain the Michaelis–Menten constant (*K_m_*), turnover number (*k_cat_*) and catalytic efficiency (*k_cat_*/*K_m_*).

The thermal stability of the ω-TA was determined by evaluating the time at which the residual activity of the enzyme was reduced to 50% of its initial activity (*t*_1/2_) and the temperature at which the enzyme activity was reduced to 50% of its initial activity (*T*_50_) subsequent to heat treatment. The half-life (*t*_1/2_) was determined by incubating the enzyme (0.2 mg/mL) in 10% (*v*/*v*) DES4 and 10% (*v*/*v*) DES7 (pH 8.0) at 40 °C for a predetermined incubation time. Consequently, the enzyme was placed on ice for 10 min after which aliquots of the enzyme were subjected to enzyme activity. The residual activity was determined and fitted on a single exponential function (LongvinMod) in Origin 8.0. On the other hand, the inactivation temperature (*T*_50_) of the enzyme in the DESs was determined by fitting of the residual activity in Boltzmann sigmoid fit post-incubation of the enzyme in the respective DESs for 10 min at a temperature range of 30–55 °C.

#### 3.2.7. Storage Stability of ω-Transaminase in DES

Aliquots of the enzyme solutions, which were incubated for several days in 10% (*v*/*v*) DES4, DES7, EG, PG and EGPG at 4 °C, were periodically used in an enzyme assay containing 10 mM PLP, 2.5 mM pyruvate and 2.5 mM 1-(*R*)-PEA in 10% (*v*/*v*) DES. The enzyme incubated in phosphate buffer was regarded as the reference. The residual activity of the enzyme post-incubation was compared with the relative activity prior to incubation.

#### 3.2.8. Product Quantification by HPLC

The analysis of the enzymatic product (i.e., acetophenone) was conducted using an Agilent1220 Infinity HPLC coupled with a C12 reverse-phase column (150 × 2.0 mm). The UV detection was conducted at 245 nm. A mobile phase comprised acetonitrile (100%) and 0.1% (*v*/*v*) formic acid in water (3:7) with an isocratic elution flow rate of 0.3 mL/min at 30 °C. The injection volume was 10 µL.

#### 3.2.9. Molecular Docking Simulation

The model was prepared on the basis of the crystal structure of transaminase with PLP (PDB ID: 4CE5), which was retrieved from the Protein Data Bank (http://www.rcsb.org, accessed on 15 January 2023). Both the A and B chains of the enzyme were considered in the computational model in which the substrate was not modified to the PLP. The protonation states of all ionizable residues were assigned via the PROPKA package [39] at pH 8.0 according to the experimental condition. The location of ChCl, EG and PG (DES7 constituents) with the enzyme during the transamination reaction were determined by the Docking Modular of YASARA. The 25 potential binding sites of the three DES7 constituents were selected for possibility and binding energy analysis to determine their effect on the catalytic activity of the enzyme.

The molecular dynamics system (MD) was solvated into a rectangular TIP3P water box [40] of 86 × 94 × 88 Å^3^ and neutralised by adding positively charged Na^+^ and negatively charged Cl^−^ counter ions. The ChCl, EG and PG were added to the system with the concentration of 10% (*v*/*v*). The initial coordinates and topology parameters were generated by YASARA in AMBER 18 [41] for MD simulation.

All model systems were optimised through MM-level energy minimisation to adjust the poor interatomic interaction. After 5000 steps of the steepest descent energy minimisation, the conjugate gradient iteration carried out 5000 cycles. The optimised system was gradually heated from 0 to 300 K at 100 ps under the NVT ensemble and then balanced at 100 ps under the NPT ensemble to relax the system density to 1.0 g/m^3^. Finally, based on the periodic boundary conditions, a 100 ns MD simulation was performed in the NPT ensemble with an integration time step of 1 fs. During the MD simulation, a cut-off value of 12 Å was set for van der Waals and electrostatic interactions. All classical MD simulations were completed using YASARA software, and the simulated trajectory was completed using PyMOL 2.0.7 software (http://PyMOL.org, accessed on 15 January 2023) for visual analysis [42].

## 4. Conclusions

The enzymatic activity of the ω-TA in 10 aqueous solutions of DES as cosolvents and the individual constituents of the most potent aqueous DES solution was investigated. In particular, the aqueous solution of the ternary-based DES (DES7) significantly improved the catalytic activity and thermal stability of the enzyme 2.4 and 2.7 times, respectively. The catalytic efficiency, turnover number, substrate affinity and stability of the enzyme in the aqueous solutions of the selected DESs were higher than in the aqueous solutions of the DES components and conventional buffer. The synergetic effects of the DES constituents, thus, attributed to an increase in catalytic efficiency, substrate affinity and stability of the transaminase. The aqueous solution of DES7 was excellent in preserving enzyme activity, as the relative activity was 118% up to day 15. Molecular docking studies illustrated that the enzyme formed some hydrogen bonding with the amino acids of the enzyme, which could have induced conformational changes on the enzyme, promoting efficient interaction between the substrate and the enzyme. The application of aqueous DES solutions as cosolvents with an appropriate HBA and HBD composition in transaminase-catalysed reactions, thus, is a promising green strategy for the efficient synthesis of chiral amines.

## Figures and Tables

**Table 1:** The deep eutectic solvents prepared with their respective molar ratios.

DES Code	Composition	Molar Ratio
DES1	Choline chloride:urea	1:1
DES2	Choline chloride:urea	1:2
DES3	Choline chloride:urea	1:3
DES4	Choline chloride:ethylene glycol	1:1
DES5	Choline chloride:glycerol	1:1
DES6	Choline chloride:1,2-propanediol	1:1:1
DES7	Choline chloride:ethylene glycol:1,2-propanediol	1:1:1
DES8	Choline chloride:glycerol:1,2-propanediol	1:1:1
DES9	Choline chloride:urea:ethylene glycol	1:1:1
DES10	Choline chloride:ethylene glycol:glycerol	1:1:1

**Table 2:** Kinetic parameters of ω-TA in aqueous solutions of DES4 and DES7 with varying substrate concentrations of 1-(*R*)-PEA. The experiment was conducted in parallel with the conventional buffer.

Medium	*K_m_^pyruvate^* (mM)	*k_cat_*^pyruvate^ (min^−1^)	*k_cat_/K_m_^pyruvate^* (L (min·mmol)^−1^)	*K_m_*^1-(*R*)-PEA^ (mM)	*k_cat_*^1-(*R*)-PEA^ (min^−1^)	*k_cat_*/*K_m_*^1-(*R*)-PEA^ (L·(min·mmol)^−1^)
Buffer	0.64 ± 0.07	32.98 ± 0.03	51.17	0.65 ± 0.07	48.16 ± 0.05	74.09
DES4	0.60 ± 0.06	40.91 ± 0.06	68.62	0.52 ± 0.04	95.48 ± 0.06	183.61
DES7	0.51 ± 0.03	45.37 ± 0.02	89.84	0.48 ± 0.02	102.87 ± 0.04	214.93
ChCl	0.97 ± 0.13	8.22 ± 0.012	8.43	0.83 ± 0.09	11.08 ± 0.09	13.31
EG	0.75 ± 0.08	19.01 ± 0.02	25.35	0.73 ± 0.09	26.83 ± 0.03	36.59
PG	0.81 ± 0.08	24.11 ± 0.08	29.88	0.79 ± 0.07	39.31 ± 0.04	49.50
EGPG	0.69 ± 0.07	26.34 ± 0.02	38.00	0.67 ± 0.03	43.62 ± 0.04	65.06

**Table 3:** The stability of the ω-TA in different solvents.

Solvent	*T*_50_ °C	*t*_½_ (min)
Buffer	38.0	10.49
DES4	40.8	20.57
DES7	42.2	28.36

**Table 4:** The docking affinity energies and intermolecular interactions between the HBDs of DES7 and key amino acid residues, as illustrated by PyMOL and Discovery Studio 4.0.

HBD	Docking Energy (kcal mol^−1^)	Interaction	From	To	Distance (Å)
EG	−9.5	Hydrogen bond	EG	Lys180	2.9
			Tyr60	EG	2.0
		Carbon hydrogen bond	Val62	EG	2.9
			EG	His55	2.2
PG	−11.4	Hydrogen bond	PG	His55	2.1
			Tyr60	PG	2.2

## Data Availability

The data from the research are contained within the manuscript and Supplementary Materials; further inquiries can be directed to the corresponding author.

## Supplementary Materials

The following supporting information can be downloaded at: https://www.mdpi.com/article/10.3390/molecules28093895/s1, Table S1: The factors and response used to plot the three-dimensional response surface plots; Table S2: The analysis of variance (ANOVA) for the quadratic model of the enzymatic activity of the ω-TA; Figure S1: HPLC analysis of the enzymatic product; Figures S2 and S3: Determination of the kinetic parameters of the ω-TA; Figure S4: Docking poses of enzyme with EG and PG at the lowest absolute affinity values.
