# Supplementary material for: Improved Stability and Catalytic Efficiency of ω-Transaminase in Aqueous Mixture of Deep Eutectic Solvents
Source: Molecules. 2023 May 5;28(9):3895. doi: 10.3390/molecules28093895 (PMC10180074; doi:10.3390/molecules28093895)
Supplement: Supplementary file 1 [file molecules-28-03895-s001.zip › molecules-2338054-supplementary.pdf]

Improved Stability and Catalytic Efficiency of ω-Transaminase in Aqueous Mixture of Deep Eutectic Solvents

Hongpeng Wang[1,†], Mercy V. Masuku[1,†], Yachen Tao[1], Jiayao Yang[1], Yi Kuang[2], Changjiang Lyu[1], Jun Huang[1*], Shengxiang Yang[2*]

[1.]Zhejiang Provincial Key Lab for Chemical and Biological Processing Technology of Farm Product, School of Biological and Chemical Engineering, Zhejiang University of Science and Technology, Hangzhou 310023, China.

[2.] Zhejiang Provincial Key Laboratory of Chemical Utilization of Forestry Biomass, Zhejiang A&F University, Hangzhou 311300, China.

*Corresponding author:

Jun Huang

E-mail: huangjun@zust.edu.cn

Shengxiang Yang

E-mail: shengxiangyang2000@zafu.edu.cn

† These authors contributed equally to this work.

Table S1. The factors and response used to plot the three-dimensional response surface plots.

**Standard**	**Run**	**Factor 1 A: Concentration (%)**	**Factor 2 B: pH**	**Factor 3 C: Temperature (°C)**	**Response 1 Specific activity (U/mg)**
13	1	10.00	8.00	40.00	2.61232
9	2	10.00	7.00	35.00	1.20977
16	3	10.00	8.00	40.00	2.47542
8	4	20.00	8.00	45.00	0.70641
10	5	10.00	9.00	35.00	1.03529
6	6	20.00	8.00	35.00	1.27386
1	7	0.00	7.00	40.00	1.32237
12	8	10.00	9.00	45.00	0.678203
17	9	10.00	8.00	40.00	2.65764
4	10	20.00	9.00	40.00	0.742966
14	11	10.00	8.00	40.00	2.53591
11	12	10.00	7.00	45.00	1.13618
5	13	0.00	8.00	35.00	0.76295
7	14	0.00	8.00	45.00	0.890177
15	15	10.00	8.00	40.00	2.66573
2	16	20.00	7.00	40.00	0.323813
3	17	0.00	9.00	40.00	0.20329

Table S2. The analysis of variance (ANOVA) for the quadratic model of the enzymatic activity of the ω-TA.

**Source**	**Sum of Squares**	**Degree of Freedom**	**Mean Square**	**F-Value**	***p*-value Prob > F**	**Significance Level**
Model	11.93	9	1.33	88.63	<0.0001	significant
A-DES Conc	2.169E-003	1	2.169E-003	0.15	0.7146	
B-pH	0.22	1	0.22	14.84	0.0063	
C-Temp	0.095	1	0.095	6.34	0.0399	
AB	0.59	1	0.59	39.56	0.004	
AC	0.12	1	0.12	8.07	0.0250	
BC	0.020	1	0.020	1.34	0.2844	
A^2	4.41	1	4.41	295.21	<0.0001	
B^2	3.54	1	3.54	236.98	<0.0001	
C^2	1.82	1	1.82	121.60	<0.0001	
Residual	0.10	7	0.015			
Lack of Fit	0.078	3	0.026	3.86	0.1123	not significant
Pure error	0.027	4	6.715E-003			
Cor Total	12.03	16				
R^2			0.9913			
Adj R^2			0.9801			
			26.400			

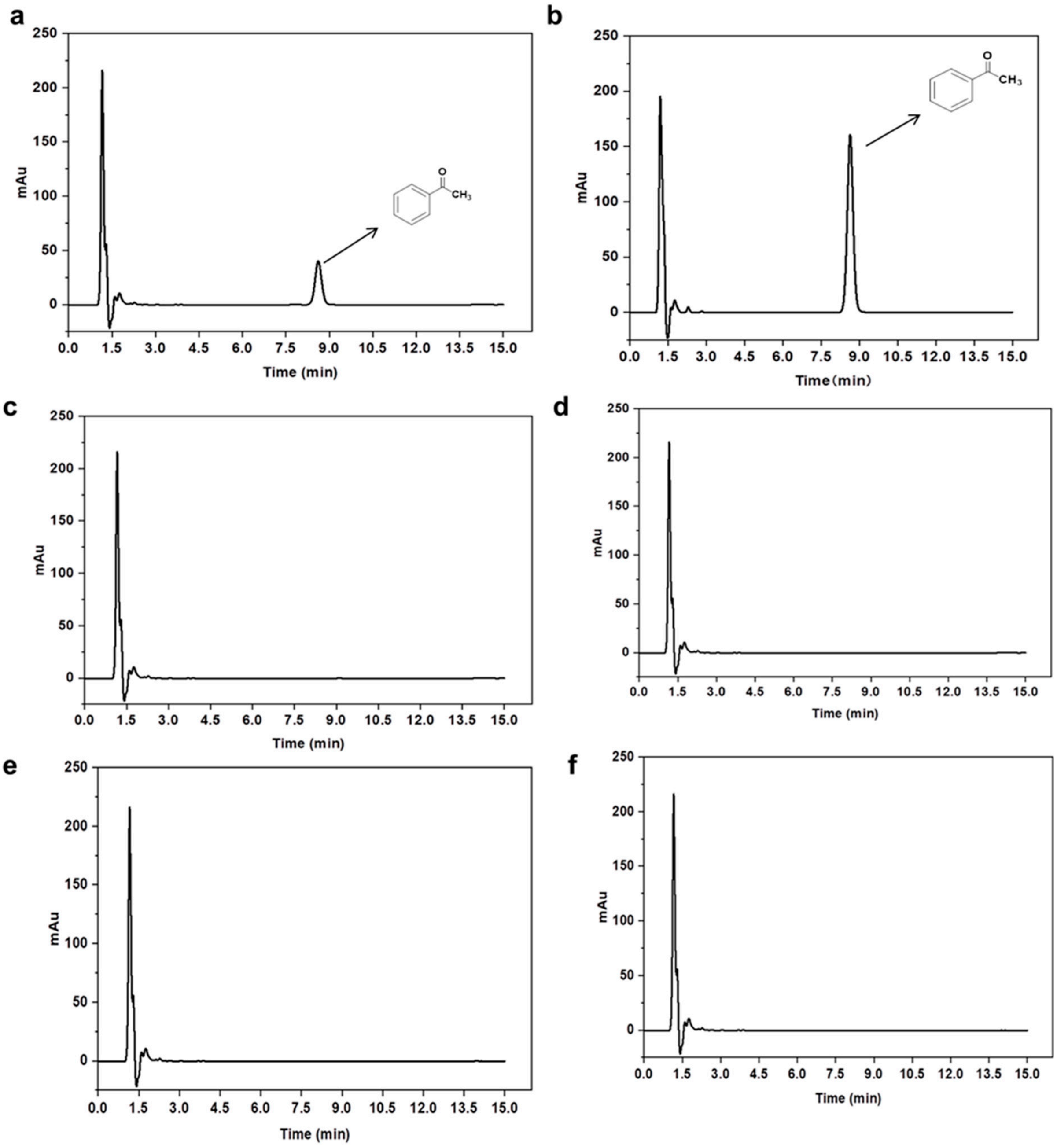

Figure S1. HPLC analysis of the enzymatic product (acetophenone). (**a**) HPLC analysis of the enzymatic product in the phosphate buffer; **(b**) HPLC analysis of the enzymatic product in aqueous deep eutectic solvent (ChCl:EG:PG); (**c**) HPLC analysis of the sample containing aqueous solution of ChCl:EG:PG without the enzyme; (**d**) HPLC analysis of the sample containing ChCl without the enzyme; (**e**) HPLC analysis of the sample containing aqueous solution of EG without the enzyme (**f**) HPLC analysis of the sample containing aqueous solution of EG without the enzyme.

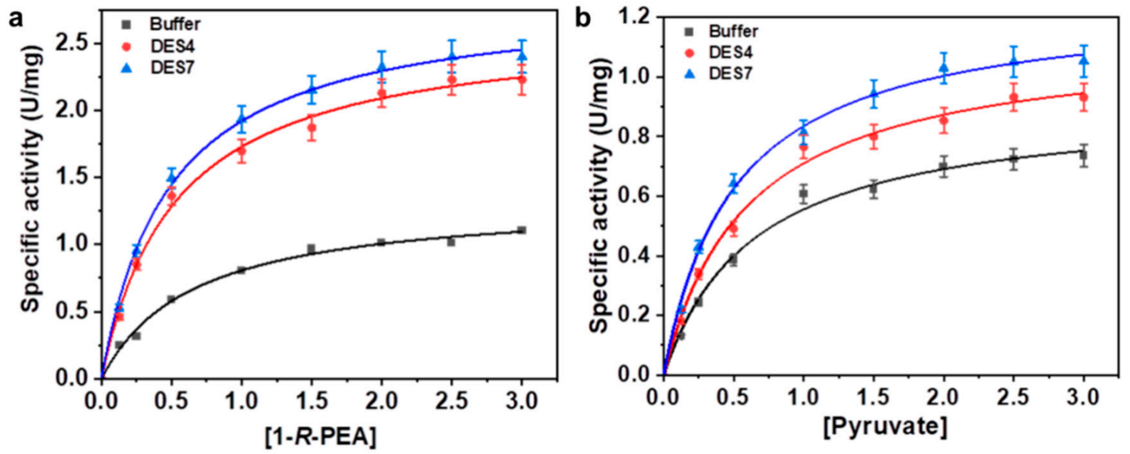

Figure S2. Determination of the kinetic parameters of the ω-TA by varying 1-*R*-PEA concentration (**a**) and varying pyruvate concentation (**b**).

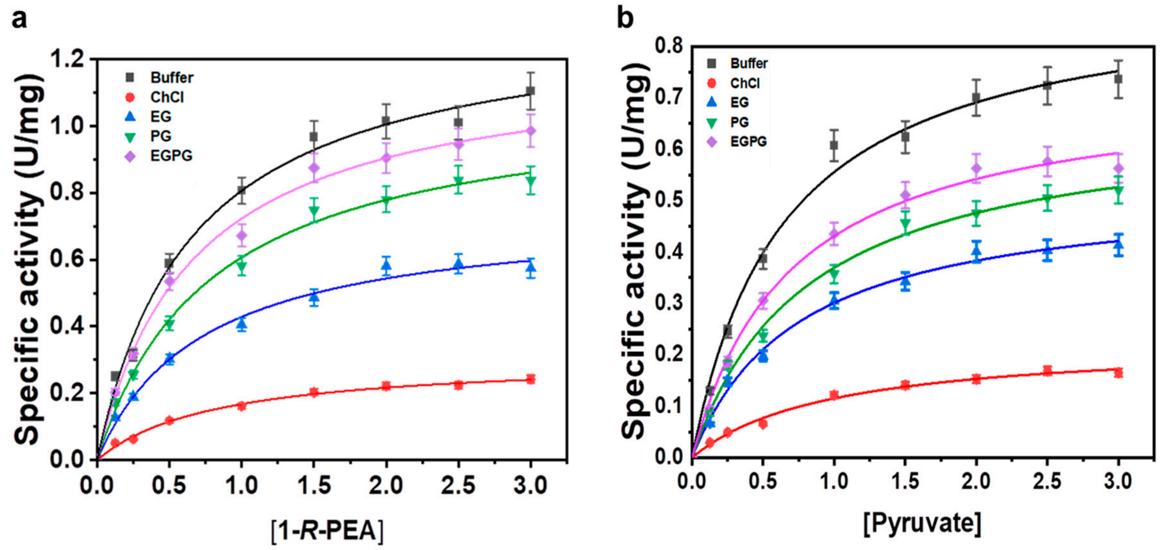

Figure S3. Determination of the kinetic parameters of the ω-TA with 10% (v/v) DES constituents using varying 1-*R*-PEA (**a**) and pyruvate (**b**).

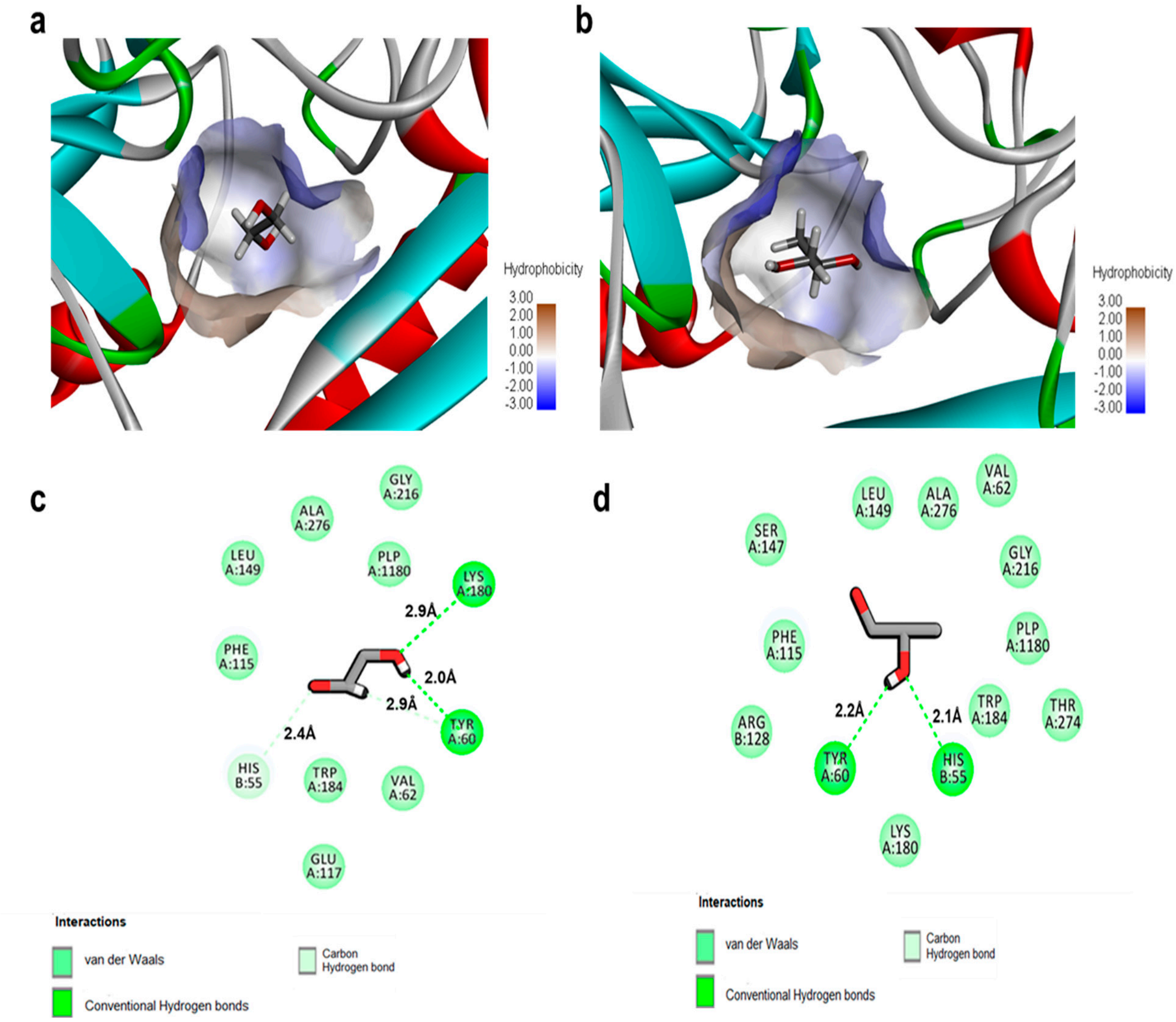

Figure S4. (**a**) Docking poses of enzyme with EG and PG; (**b**) at the lowest absolute affinity values. (**c**) Molecular diagrams of the interaction between EG and PG (**d**) with amino acid residues.
